# Manganese–Zinc Synergy in Prussian Blue Analogues for Long-Cycle Aqueous Zinc-Ion Battery Cathodes

**DOI:** 10.3390/nano16100617

**Published:** 2026-05-17

**Authors:** Jiangtao Pan, Yiyuan Yang, Xiaodong Liang, Qian Zhang, Junqing Huang, Debing Long, Xiyan Bao, Luyang Ge, Xiaolin Wu, Houzhao Wan

**Affiliations:** 1 School of Physics and Electronic-Information Engineering, Hubei Engineering University, Xiaogan 432000, China; 2 Hubei Key Laboratory of Micro-Nanoelectronic Materials and Devices, School of Integrated Circuits, Hubei University, Wuhan 430062, China

**Keywords:** aqueous rechargeable zinc-ion batteries, Prussian blue analogues, cathode materials, electrochemical stability, zinc hexacyanoferrate

## Abstract

Aqueous zinc-ion batteries (AZIBs) are regarded as promising electrochemical energy storage devices owing to their low cost, intrinsic safety, abundant zinc reserves, and desirable specific capacity. Prussian blue analogues (PBAs) have been extensively investigated because of their inexpensive raw materials, ease of fabrication, open frameworks, and high theoretical specific capacity; however, the application of PBAs as cathode materials for aqueous zinc-ion batteries (AZIBs) is hindered by poor cycling performance and limited capacity. In this work, a small amount of manganese ions was successfully introduced into the N-coordinated metal sites of zinc hexacyanoferrate (ZnHCF) to tailor its electrochemical stability. The N-coordinated metal species in PBAs directly influence the intercalation chemistry of Zn ions. The coexistence of manganese and zinc in manganese-substituted zinc hexacyanoferrates (MZHCFs) generates a synergistic effect that suppresses Jahn–Teller distortion and cathode material dissolution, endowing MZHCFs with superior cycling performance compared with PBAs containing a single N-coordinated metal (Mn or Zn). At a Mn content of 10%, a specific discharge capacity of 100 mAh g^−1^ is achieved at a current density of 1 A g^−1^, and the capacity retention is optimized, showing no decay relative to the initial discharge capacity after 2000 galvanostatic cycles. This study demonstrates that substituting the N-coordinated metal in PBAs with other metal ions is an effective strategy to improve their electrochemical cycling stability and capacity.

## 1. Introduction

While lithium-ion batteries (LIBs) and sodium-ion batteries (NIBs) have dominated the energy storage landscape due to their high energy densities and mature intercalation chemistry, the search for next-generation systems continues [1,2]. For instance, the development of advanced anodes, such as scalable microwave-assisted Ti_3_C_2_T_x_ MXenes, has significantly enhanced the rate performance and structural stability of LIBs and NIBs [3]. Similarly, intercalating Fe_3_O_4_ nanoparticles into reduced graphene oxide (rGO) has been shown to bridge the gap between high-energy LIBs and high-power supercapacitors [4]. However, despite these advancements in non-aqueous and organic systems, concerns regarding the scarcity of lithium resources, high costs, and intrinsic safety risks (e.g., flammability) remain persistent.

In this context, Aqueous Rechargeable Zinc-Ion Batteries (ARZIBs) have emerged as a highly competitive alternative. They offer inherent safety through the use of non-flammable aqueous electrolytes, alongside the high abundance and high theoretical capacity (820 mAh g^−1^) of zinc metal anodes [5,6,7,8,9]. However, the development of ARZIBs is primarily hindered by the lack of cathode materials that can simultaneously provide high reversible capacity and long-term cycling stability. Conventional cathode materials often suffer from structural collapse and active material dissolution during the repeated intercalation/extraction of divalent Zn^2+^ ions, which exhibit strong electrostatic interactions with the host lattice [10,11,12,13,14,15].

So far, a wide variety of materials have been reported as cathodes for aqueous rechargeable zinc-ion batteries (ARZIBs), including manganese-based oxides, vanadium-based materials, Prussian blue analogues (PBAs), Chevrel phases, polyanionic compounds, metal dichalcogenides, and organic compounds [16,17,18,19,20,21,22,23,24,25]. Among these, Prussian blue analogues (PBAs) with open-framework structures have drawn intense research attention owing to their simple preparation, good stability, non-toxic electrolytes, low cost, and structural diversity [25,26,27,28,29,30]. They possess a three-dimensional porous framework structure that facilitates the rapid and reversible electrochemical insertion/extraction of various cations, including zinc ions [22,31,32,33,34,35,36,37,38,39]. All these features make PBAs a highly promising cathode material for ARZIBs. However, PBAs suffer from problems such as unstable electrochemical performance and poor cycle life. In ARZIBs, this instability can be attributed to drastic structural and compositional transformations and the dissolution of active materials [24].

To address the electrochemical instability of PBA cathode materials and improve their cycle life, researchers have adopted strategies such as high-entropy design, defect engineering, and doping engineering [40,41,42,43]. For example, Xing et al. [44] introduced five transition metal elements (Mn, Co, Ni, Fe, and Cu) into the N-coordinated M-lattice to prepare a high-entropy Prussian blue analogue (HE-PBA). The HE-PBA exhibited enhanced cycling stability and capacity retention, maintaining a specific capacity of around 80 mAh g^−1^ after 100 cycles at a current density of 0.1 A g^−1^. Fu et al. [45] successfully synthesized a core–shell-structured Mn@Fe_1_-PBA through chemical regulation, which displayed outstanding electrochemical performance, delivering a high capacity of 119.2 mAh g^−1^ after 100 cycles at 1 A g^−1^, making it a highly promising cathode material for AZIBs. Pan et al. [46] designed various Zn-substituted MnHCFs, among which Mn_0.8_Zn_0.2_HCF demonstrated the best cycling stability and rate capability, achieving a capacity retention of 73.2% after 1000 cycles at 850 mA g^−1^, significantly surpassing that of MnHCF (33.2%). Zhang et al. [47] successfully synthesized a Zn-substituted FeHCF composite material via a typical co-precipitation method, which exhibited a high initial discharge capacity of 145.0 mAh g^−1^ at 20 mA g^−1^ and retained a considerable capacity of 73.8 mAh g^−1^ after more than 500 cycles at 1 A g^−1^. Clearly, metal ion substitution is an effective strategy to improve the capacity and capacity retention of zinc batteries, yet metal ion substitution based on zinc hexacyanoferrate (ZnHCF) still requires further systematic investigation.

Herein, trace Mn cations were successfully incorporated into nitrogen-linked metallic nodes of zinc hexacyanoferrate frameworks using a straightforward wet-chemical precipitation protocol, resulting in an exceptionally robust working electrode. Systematic investigation reveals that the concurrent presence of both transitional elements within the substituted host lattice triggers cooperative electronic interactions. This mutual reinforcement effectively mitigates severe Jahn–Teller structural degradation and suppresses transition-metal dissolution during electrochemical operations, thereby granting the composite high-rate cyclability far exceeding that of Prussian blue counterparts relying on solitary nitrogen-bonded metallic species. Specifically, optimizing the substitution level to a decimal fraction (10%) delivers a specific discharge capability of 100 mAh g^−1^ under a current density of 1 A g^−1^. Remarkably, this optimal configuration maintains its initial capacity completely without any measurable deterioration across 2000 continuous charging loops, contrasting sharply with the pristine matrix which preserves merely 36% under identical conditions. Consequently, these multi-component coordination networks emerge as highly prospective candidates for aqueous zinc-based devices, offering a viable pathway toward efficient, ecological energy storage technologies.

## 2. Materials and Methods

### 2.1. Synthesis of ZnHCF

Sinopharm Chemical Reagent Co., Ltd. (Shanghai, China) supplied the starting precursors, namely ZnSO_4_, K_3_Fe(CN)_6_, and Mn(NO_3_)_2_. Prussian blue analogues (PBAs) were prepared through a straightforward liquid-phase precipitation strategy. In a representative fabrication process targeting pristine zinc hexacyanoferrate (ZnHCF), two millimoles of ZnSO_4_ was thoroughly dispersed within 100 mL of purified deionized water, yielding precursor solution A. Concurrently, K_3_Fe(CN)_6_ (2 mmol) was dissolved under identical solvent conditions to generate the corresponding solution B. Under rapid magnetic agitation, solution B received a dropwise addition of solution A throughout a 1 h duration. This reacting system was continuously stirred at ambient temperature for 12 h, followed by a subsequent static aging stage lasting 6 h. Finally, the solid precipitate was isolated via centrifugal separation, subjected to triple washing cycles using deionized water, and subsequently dehydrated overnight (12 h) at 70 °C.

### 2.2. Synthesis of MZHCF

To obtain pure MnHCF, Mn(NO_3_)_2_ was utilized as a direct substitute for the zinc precursor. Meanwhile, bimetallic MZHCF networks were fabricated through an identical wet-chemical route, employing co-dissolved precursor formulations containing both transition-metal cations. Based on the feed stoichiometry of the active species, where the nominal Mn^2+^/Zn^2+^ ratios were adjusted to 0%, 5%, 10%, 15%, and 20%, the resulting coordination frameworks were systematically categorized under the shorthand notation of MZHCF-x (with x denoting the respective manganese percentage as 0, 5, 10, 15, and 20).

### 2.3. Characterization

To resolve the crystallographic and morphological attributes of the synthesized specimens, field-emission scanning electron microscopy (FESEM; JSM 7100F platform, JEOL Ltd., Tokyo, Japan) and focused-ion-beam scanning electron microscopy (FIB-SEM; Zeiss CrossBeam 540, Carl Zeiss AG, Oberkochen, Germany) were conducted. Phase constitutions were identified using an X-ray diffractometer (XRD; D8 Advance, Bruker AXS, Karlsruhe, Germany), whereas surface elemental environments were mapped via X-ray photoelectron spectroscopy (XPS; ESCALAB 250Xi, Thermo Fisher Scientific, East Grinstead, UK). For high-resolution internal structure and localized chemical mapping, transmission electron microscopy (TEM; JEM-2100F, JEOL Ltd., Tokyo, Japan) coupled with energy-dispersive X-ray spectroscopy (EDX) was employed. Quantitative elemental stoichiometries of the bulk materials were determined using inductively coupled plasma optical emission spectrometry (ICP-OES; PlasmaQuant PQ9000, Analytik Jena, Jena, Germany). To evaluate long-term electrochemical durability, the fabricated cathode (serving as the working electrode) underwent 2000 continuous galvanostatic charge–discharge loops inside a three-electrode cell configuration containing a defined volume of 1 M ZnSO_4_ + 1 M MnSO_4_ liquid medium. Upon concluding this degradation test, the entire electrolyte reservoir was vigorously agitated to achieve uniform ion distribution, and an aliquot was subsequently extracted for post-test ICP-OES analysis. Additionally, vibrational characteristics of chemical bonds were recorded using Fourier transform infrared spectroscopy (FTIR; Nicolet iS50, Thermo Fisher Scientific, Madison, WI, USA).

### 2.4. Electrochemical Measurements

The synthesized electroactive hosts (ZnHCF or the substituted MZHCF counterparts), conductive carbon black (Super P), and polyvinylidene difluoride binder (PVDF) were thoroughly blended in a dry mass proportion of 7:2:1. To ensure absolute homogeneity, the combined powders underwent manual dry-grinding inside a high-purity agate-based mortar. This homogenized mixture was subsequently suspended in an appropriate volume of 1-methyl-2-pyrrolidone NMP under intensive magnetic stirring to form a stable slurry. Thereafter, the fluid paste was uniformly cast upon a conductive stainless-steel substrate, dried under vacuum at 60 °C for a 12 h period, and eventually sectioned into circular plates (9 mm in diameter) to construct the individual working electrodes. The average mass loading of the active components was regulated at approximately 1.5 mg per disc. Electrochemical evaluation utilizing galvanostatic charge–discharge cycling was conducted inside CR2025-type coin-cell configurations (Guangdong Tob New Energy Technology Co., Ltd., Dongguan, China). These testing devices were assembled by pairing the prepared working electrode with a metallic zinc counter-electrode possessing a diameter of 12 mm and a thickness of 30 μm, partitioned by an industrial NKK-MPF30AC-100 separating membrane (NKK-MPF30AC-100, Nippon Kodoshi Corp., Kochi, Japan). The liquid transport medium employed throughout the measurements consisted of a mixed aqueous electrolyte containing 1 M ZnSO_4_ and 1 M MnSO_4_. To clarify the roles of structural Mn and electrolyte Mn^2+^ additives, control experiments were conducted for ZnHCF and MZHCF-10 with or without MnSO_4_ (Figure S4). In the Mn-free electrolyte, MZHCF-10 undergoes a capacity drop from ~118 to ~95 mAh g^−1^ within 20 cycles due to lattice Mn leaching driven by the concentration gradient (Figure S4b). The addition of MnSO_4_ effectively suppresses this dissolution via the common-ion effect, maintaining a stable capacity. Crucially, MnSO_4_ addition accelerates the capacity decay of pristine ZnHCF (Figure S4a), refuting the hypothesis that dissolved Mn^2+^ merely undergoes surface adsorption or parasitic reactions to simulate stability. These results confirm that structural Mn substitution is the intrinsic driver for the zero-decay behavior, while electrolyte Mn^2+^ serves as a necessary thermodynamic partner to preserve framework integrity.

To evaluate the redox kinetics, cyclic voltammetric (CV) scans were performed using a CHI 760E electrochemical analyzer (CHI 760E, Shanghai Chenhua Instrument Co., Ltd., Shanghai, China). These investigations were configured in a Swagelok-design three-electrode cell (Swagelok Company, Solon, OH, USA), utilizing an auxiliary zinc plate alongside a reference SCE system. Constant-current charge–discharge (GCD) evaluations were executed utilizing a Landt CT3001A battery-testing platform (CT3001A, Wuhan Lanhe Electronics Co., Ltd., Wuhan, China), sweeping a potential window from 1.0 to 2.0 V (versus Zn/Zn^2+^) inside a thermal incubator pre-set to 25 °C.

## 3. Results

**Figure 1:**
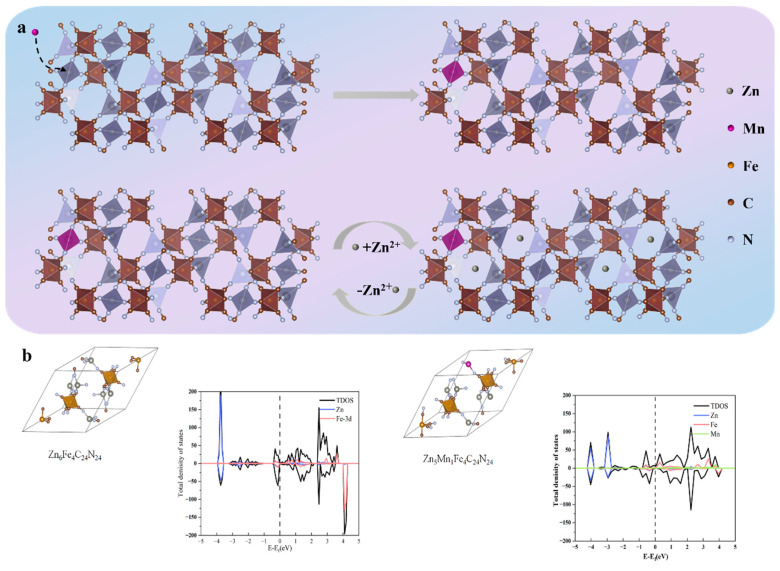
Schematic diagram of the mechanism of Mn ion substitution for Zn ions in ZnHCF and the electrochemical reaction process of MZHCF (**a**), Crystal structures of ZnHCF and MZHCF and their density of states (DOS) diagrams (**b**).

Figure 1a schematically depicts the insertion of Mn dopants into the parent ZnHCF framework via a facile liquid-phase precipitation process. These incoming Mn cations preferentially occupy the octahedral void spaces within the host lattice, strategically replacing Zn species situated at nitrogen-coordinated nodes. The highly open, three-dimensional skeletal architecture, characterized by its expansive internal cavities, delivers frictionless transport pathways and robust storage space to facilitate the reversible insertion and extraction of zinc-ion charge carriers during electrochemical operations. Manganese doping was strategically implemented to systematically probe how nitrogen-bonded metallic nodes modulate zinc-storage kinetics, while simultaneously bolstering structural and electrochemical durability. Consequently, a library of bimetallic MZHCF networks featuring varied stoichiometry was thoroughly evaluated as positive electrodes for aqueous rechargeable zinc-ion batteries, or ARZIBs, unraveling the precise correlation between dopant concentration and crystallographic structure.

**Figure 2:**
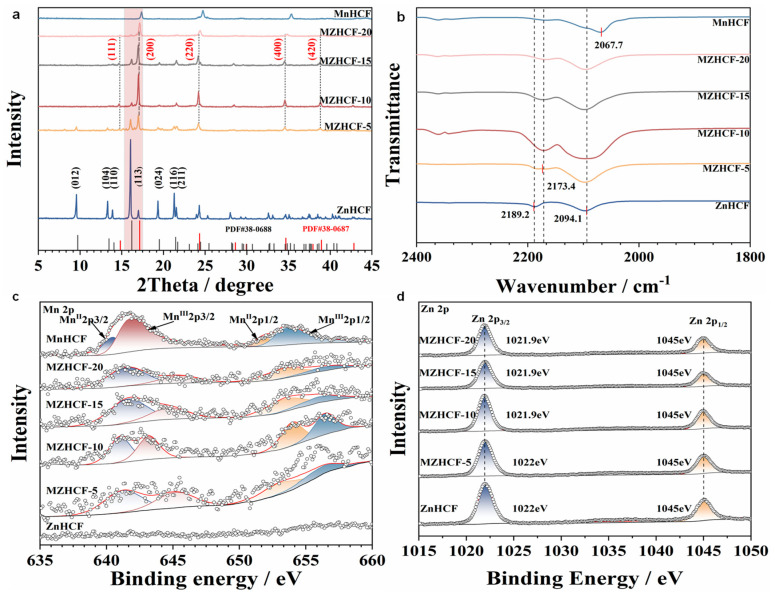
XRD patterns (**a**) and FT-IR spectra (**b**) of the ZnHCF, MZHCFs, and MnHCF samples. Mn 2p (**c**) and Zn 2p (**d**) XPS spectra of the ZnHCF, MZHCFs, and MnHCF samples.

To computationally verify the crystal structures of manganese Prussian blue (MZHCF-10) and zinc Prussian blue (ZnHCF), density of states (DOS) calculations were performed, as shown in Figure 1b. The figure presents the crystal structures and corresponding DOS plots of two materials with different chemical compositions, Zn6Fe4C24N24 and Zn5MnFe4C24N24. The left side displays the three-dimensional crystal structures, while the right side shows the total density of states and the electronic contributions of individual elements. In the DOS plot, the peaks near the Fermi level (Ef) are predominantly contributed by Fe-3d and Mn-3d orbitals. This implies that during charge/discharge, electrons can be readily transferred into or out of these d orbitals, thus delivering a high specific capacity. After the introduction of Mn, the density of states distribution near the Fermi level is modified. In battery applications, this typically translates to a wider voltage window or additional redox potentials, thereby enhancing the energy density. At the vertical dashed line (Fermi level, 0 eV), the density of states does not vanish and exhibits a finite intensity, indicating that the material possesses good intrinsic electronic conductivity. For energy storage applications, this means that charges can migrate rapidly within the material, reducing polarization and thus enabling high-rate operation. The increased symmetry suggests that the substitution modifies the electron spin polarization state, enhancing the electrical conductivity and thermal stability. Furthermore, the introduction of Mn leads to performance improvements, as demonstrated by XPS and XRD analyses (Figure 2). XPS analysis reveals that the introduction of Mn facilitates the valence changes of Mn ions during the electrochemical process of ZnHCF, enhancing the structural stability of the material while suppressing phase transitions. XRD analysis shows that the introduction of Mn significantly suppresses the cubic-to-rhombohedral phase transition and improves cycle life. These analytical results indicate that Mn incorporation not only improves the electronic structure of the material but may also substantially enhance its electrochemical performance, manifested by an increase in discharge capacity and enhanced cycling stability.

### 3.1. Characterization of PBAs

Crystallographic architectures of pristine ZnHCF, bimetallic MZHCFs, and MnHCFs were probed via X-ray diffraction (XRD), as depicted in Figure 2a. Each specimen demonstrates well-resolved and highly defined Bragg reflections, signifying a high degree of structural crystallinity. For unmodified ZnHCF, the recorded diffractogram reveals distinct reflections situated at 2theta angles of 9.68°, 13.42°, 14.00°, 16.16°, 19.46°, 21.42°, and 21.64°. These peaks are indexable to the (012), (104), (110), (113), (024), (116), and (211) lattice planes, respectively, of a rhombohedral symmetry matching PDF standard No. 38-688. Upon manganese substitution, the profiles of MZHCF display a coexistence of both rhombohedral and cubic phases. Specifically, the emergence of novel diffraction signals at 2theta = 14.78°, 17.08°, 24.2°, 34.60°, and 38.8° can be assigned to the (111), (200), (220), (400), and (420) miller indices of a cubic ZnHCF framework (referenced to PDF No. 38-687) [48,49]. As the manganese doping level elevates, the intensity of the cubic (200) reflection undergoes a non-monotonic evolution, initially intensifying before diminishing. Conversely, the rhombohedral (113) reflection exhibits an opposite trajectory, first weakening and subsequently recovering. Both trends display a distinct inflection threshold at MZHCF-10, signifying that the volume fraction of the cubic phase relative to the rhombohedral phase reaches its peak at this specific dopant concentration before declining.

Depending on internal hydration levels, zinc hexacyanoferrate ZnHCF typically crystallizes into dual distinct polymorphs: namely, isotropic cubic or anisotropic rhombohedral configurations. Generally, heavily hydrated networks stabilize in the cubic phase, whereas anhydrous equivalents prefer a rhombohedral geometry. Prior reports suggest that substituting zinc with manganese dopants substantially promotes the accumulation of intracrystalline water molecules [49]. Chemically, the generic formulation of this coordination framework is commonly represented as Zn_3_[Fe(CN)_6_]_2_·nH_2_O, wherein n > 0 stabilizes the cubic polymorph, while a zero value (n = 0) defines the dry rhombohedral state. Given a nominal Zn/Fe stoichiometry of 1.5, nearly one-third of the [Fe(CN)_6_]^3−^ ligand nodes within the cubic sublattice remain unoccupied, leaving structural vacancies that are subsequently filled by coordinated aqua ligands. Within this cubic lattice, the zinc cations reside in a hexacoordination sphere, linking with four nitrogen-coordinated cyanometallate groups and a pair of aqua ligands. Conversely, the zinc centers inside this rhombohedral polymorph assume a tetracoordinated state, being exclusively coordinated by four surrounding Fe(CN)_6_ complexes. As shown by the thermogravimetric analysis in Figure S1, the water content increases after Mn substitution, indicating that Mn substitution increases the lattice water content, which structurally stabilizes the cubic framework and suppresses the transition to the anhydrous rhombohedral phase during cycling.

Fourier-transform infrared (FT-IR) profiles are presented in Figure 2b. Regarding the unmodified ZnHCF specimen, the absorption band centered at 2094.1 cm^−1^ is assigned to stretching vibrations of cyano groups within the FeII–CN–ZnII linkages. Concurrently, the shoulder located near 2189.2 cm arises from the stretching modes of FeIII–CN–ZnII coordination units within the rhombohedral lattice framework [50]. As manganese incorporates progressively, an additional vibrational signature emerges at approximately 2173.4 cm^−1^, which is characteristic of cyanide stretching modes within cubic FeII-CN-Zn II coordination domains. The amplitude of this specific infrared reflection undergoes a parabolic trajectory, initially intensifying prior to subsequent attenuation. This spectroscopic evolution closely mirrors the phase-transition trend evidenced by the XRD results. Such behavior confirms that the relative concentration of the cubic phase experiences an initial expansion followed by a reduction as the manganese ratio rises, establishing a clear turning threshold at the MZHCF-10 composition.

X-ray photoelectron spectroscopy (XPS) measurements were performed to scrutinize the electronic impact of manganese substitution (Figure 2c,d). The pristine MnHCF counterpart displays a doublet of diffuse peaks positioned at approximately 641.2 and 653 eV, which represent the 2p_3/2_ and 2p_1/2_ spin–orbit states of manganese, respectively. In the MZHCF-10 sample, a distinct emission line emerges, arising at approximately 641.1 eV; this feature is indexable to the Mn2p_3/2_ state and remains completely missing within unmodified ZnHCF. Relative to the pure ZnHCF baseline, the Zn 2p emission envelope of MZHCF-10 undergoes a minor negative shift of about 0.1 eV. This marginal chemical displacement is likely driven by the lower electronegativity of manganese (1.55) relative to zinc (1.65), which subsequently perturbs the local electron density around the bridging CN^−^ groups. Additionally, both the Mn and Fe core-level profiles (presented in Figure S2) present coexisting oxidation states, which closely corroborates the FT-IR deductions [51]. These diagnostic signatures collectively substantiate the successful incorporation of manganese cations into the host ZnHCF lattice.

To further clarify the redox mechanism, the Fe 2p spectra (Figure S1) were analyzed. Both ZnHCF and MZHCF-10 display characteristic peaks for Fe^II^ and Fe^III^, confirming the mixed-valence state of the C-coordinated iron. Notably, the Fe^III^ peaks in MZHCF-10 exhibit a slight shift toward higher binding energy (723.8 eV for Fe^III^2p_1/2_) relative to ZnHCF (723.6 eV). This shift implies that the substitution of N-coordinated Zn by Mn modulates the electronic environment of the entire M-CN-Fe backbone, effectively tuning the Fe^2+/3+^ redox behavior and facilitating a stable solid-solution reaction pathway.

**Figure 3:**
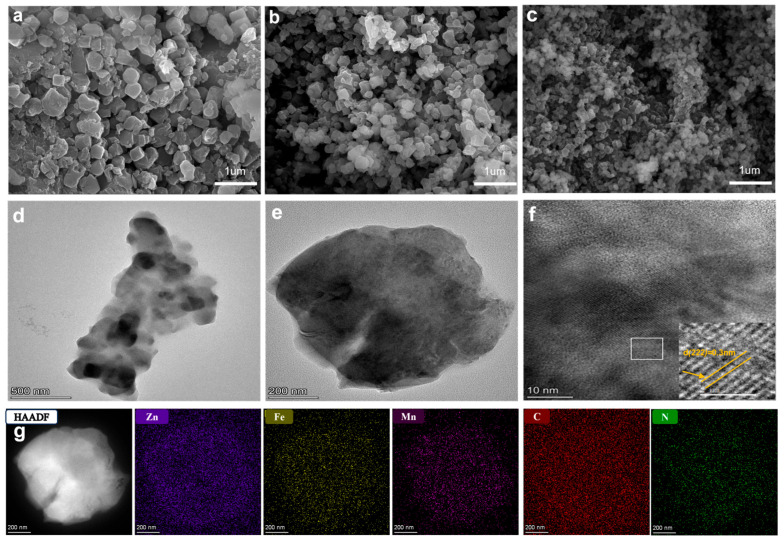
SEM images of the ZnHCF (**a**), MZHCF-10 (**b**), and MnHCF (**c**) samples. TEM images (**d**–**f**) and EDS maps (**g**) for Zn, Fe, Mn, C and N of MZHCF-10.

The spatial morphology of different specimens was resolved utilizing scanning electron microscopy (SEM), as illustrated in Figure 3a–c alongside Figure S3a–c. The unmodified ZnHCF consists of well-defined nanocubes, whose dimensions span from 230 up to 650 nm. Upon introducing manganese, the resulting MZHCF variants preserve this cubic geometry, demonstrating highly polished boundaries and average diameters within a 220–700 nm range, showing no obvious morphological variations across the diverse doping concentrations. In sharp contrast, the pure MnHCF derivative adopts a hollow-like, concave microcubic configuration, exhibiting localized dimensions scaled down to 150–300 nm. To inspect the atomic-scale details, transmission electron microscopy (TEM) was implemented (Figure 3d–f), clarifying the clear crystalline planes of the MZHCF-10 composite. Specifically, the interplanar distance measured at 0.3 nm (Figure 3f) matches the (222) crystallographic plane of the host ZnHCF framework. Energy-dispersive X-ray spectroscopy (EDS, mapping displayed in Figure 3g along with Figure S2d) was conducted to map the chemical constituents of MZHCF-10. The spectroscopic profiles verify the coexistence of zinc, manganese, iron, potassium, carbon, nitrogen, and oxygen, all of which are distributed with high homogeneity throughout the microstructural matrix. Furthermore, inductively coupled plasma (ICP) spectroscopy (summarized in Table S1) was utilized to quantify the bulk atomic ratios of potassium, zinc, and iron alongside manganese species in both unmodified and doped lattices. By normalizing the stoichiometric index of iron to unity, the exact molecular formulas of the respective systems were determined (Table 1).

### 3.2. Electrochemical Behavior of PBAs

**Figure 4:**
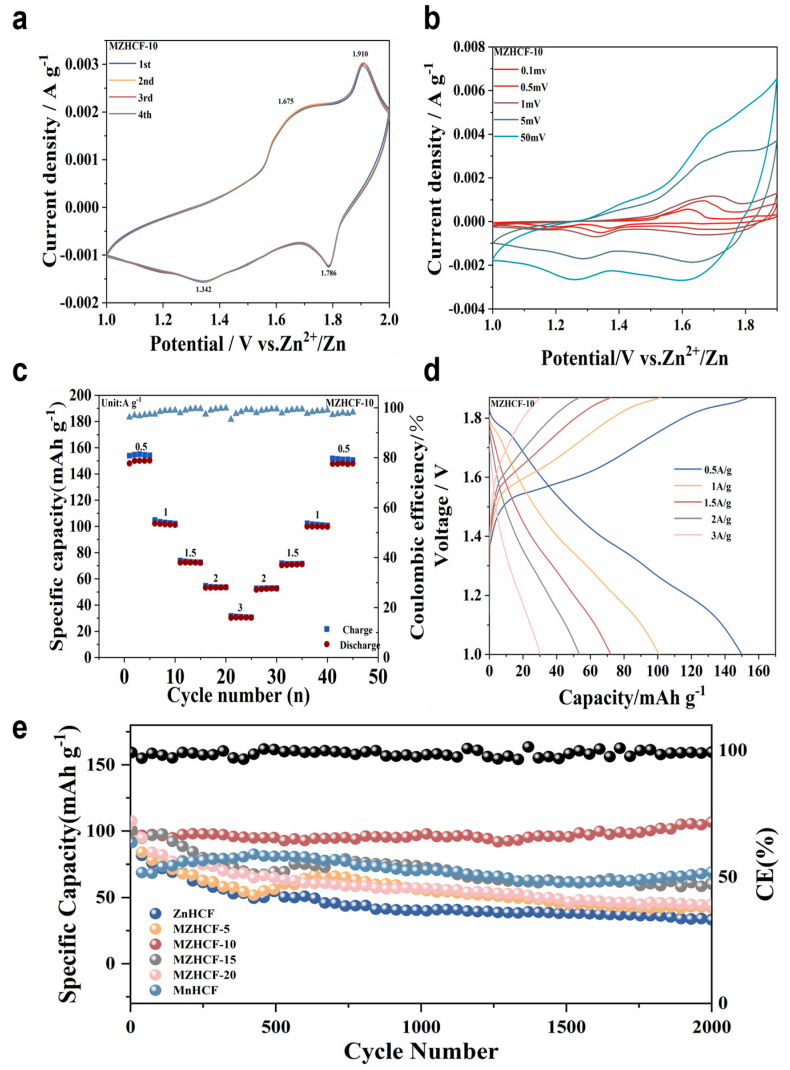
CV curves of ZnHCF (**a**), MZHCF-10 at different scanning rates (**b**). Rate capability of MZHCF-10 (**c**). Galvanostatic charge/discharge (GCD) curves of MZHCF-10 (**d**). at different current densities. Cycle performance of ZnHCF and MZHCFs at 1 A g^−1^ (**e**).

Figure S4a and Figure 4a present the respective cyclic voltammetry (CV) profiles of the pristine ZnHCF and modified MZHCF-10 electrodes. The unmodified ZnHCF anode displays two distinct anodic waves situated at 1.645 V and 1.947 V, complemented by dual cathodic counterparts at 1.333 V and 1.731 V. During the initial activation scan, the lower-potential redox features (anodic: 1.645 V; cathodic: 1.333 V) appear faint. Although these signals progressively intensify throughout successive cycles, their current responses remain subordinate compared to the higher-potential peak pair. This entire set of redox signatures is attributed to the highly reversible insertion and extraction of zinc-ion charge carriers within the ZnHCF host, matching the galvanostatic charging–discharging (GCD) traces (Figure S3c). Drawing upon literature insights, Tolentino and co-workers [52] elucidated that such dual-redox behavior in hexacyanoferrates mimics the phase-transition and solid-solution dynamics observed during sodium-ion storage. In this framework, the high-voltage electrochemical reaction is driven by a distinct first-order phase transition, whereas the lower-voltage process is governed by a single-phase solid-solution mechanism. The voltammetric response of the MZHCF-10 electrode shares general features with the baseline matrix, exhibiting anodic peaks at 1.675 V and 1.910 V, alongside cathodic peaks at 1.342 V and 1.786 V. Notably, the peak magnitudes display immediate stabilization without any noticeable activation stage. Upon continuous sweeps, the 1.910 V anodic peak dominates over the 1.675 V feature, whereas the 1.342 V peak becomes highly pronounced relative to the 1.786 V peak during the corresponding reduction sweep. These distinct electroactive regions at elevated voltage (1.910/1.786 V) and diminished voltage (1.675/1.342 V) can be reasonably assigned to the faradaic reactions of nitrogen-bonded Mn^2+/3+^ centers and carbon-bonded Fe^2+/3+^ complexes, respectively. The potential positions of these voltammetric peaks correlate exceptionally well with the galvanostatic discharge voltage plateaus of MZHCF-10 (Figure 4d). Relative to the pristine matrix, the current densities of both reduction peaks undergo considerable enhancement. This suggests that the incorporated manganese dopants effectively deter the structural phase transition while simultaneously promoting a dominant solid-solution mechanism, a behavior that closely aligns with the observed GCD plateau profiles.

To probe the electrochemical kinetics, rate-dependent cyclic voltammetric (CV) responses of the MZHCF-10 system are depicted within Figure 4b. Upon progressively accelerating the sweep rates, the anodic waves steadily migrate toward more positive potentials, whereas the cathodic equivalents shift in the negative direction, highlighting a predictable expansion of internal ohmic and kinetic polarization. To benchmark the electroactive performance, the CV signatures of pristine ZnHCF and substituted MZHCF networks were systematically evaluated under a uniform sweep velocity of 1 mV s^−1^ (Figure S4b). Strikingly, the MZHCF-10 configuration outperforms the other compositions by displaying a vastly superior current density. This elevated current output is in excellent agreement with both the galvanostatic charge–discharge (GCD) profiles and the long-term cycling trends (Figure 4e), unambiguously proving that this specific dopant stoichiometry yields the maximum charge-storage capacity. The EIS spectra are presented in Figure S5, the pristine ZnHCF exhibits an exceptionally large semicircle in the high-to-medium frequency region, indicating an extremely high charge-transfer resistance (Rct) and sluggish electrochemical kinetics. Remarkably, upon Mn substitution, the semicircle diameters for all MZHCF samples (MZHCF-5, 10, 15, 20) decrease drastically. This direct spectroscopic evidence unambiguously demonstrates that the substitution of Mn ions fundamentally optimizes the electronic structure of the Fe-CN-M framework, leading to significantly enhanced electrical/ionic conductivity and lowered charge-transfer resistance. Notably, while the initial EIS measurements demonstrate the kinetic enhancement from Mn substitution, EIS data after 100 and 2000 cycles were not included. This is because the accumulation of surface passivation layers (e.g., basic zinc sulfates) and the progressive polarization of the Zn anode during extended cycling introduce significant parasitic impedances. These factors mask the intrinsic charge-transfer characteristics of the active material, rendering the decoupled resistance values and equivalent circuit fitting results unreliable for rigorous scientific interpretation.

Rate capabilities of the MZHCF-10 working electrode were systematically tested across various current loads (Figure 4c). Specifically, the composite yielded specific capacities of 149.26, 105.03, 74.55, 55.52, and 33.42 mAh g^−1^ when evaluated under applied current densities of 0.5, 1.0, 1.5, 2.0, and 3.0 A g^−1^, respectively. Upon reverting the current density back to the initial 0.5 A g^−1^ level, a prominent capacity recovery of 143.78 mAh g^−1^ was achieved. This corresponds to an outstanding retention efficiency of 96.3%, confirming the superior rate tolerance of this manganese-modified cathode framework. For comparison, corresponding galvanostatic discharge profiles of the pristine ZnHCF under equivalent current ranges are compiled within Figure S3c. The discharge profiles display two distinct sloping segments within the potential windows of 1.8–1.4 V and 1.4–0.9 V (versus Zn^2+^/Zn). These features are fundamentally coupled with the structural phase reorganization and the single-phase solid-solution transition, respectively, both representing the multi-step insertion mechanism of zinc-ion guest species into the ZnHCF host lattice (Equation (1)) [53].

(1) $${\mathrm{Zn}}_{1.7}[{\mathrm{Fe}\left(\mathrm{CN}\right)}_{6}]+x{\mathrm{Zn}}^{2+}+{\;2\mathrm{xe}}^{-}\to {\mathrm{Zn}}_{1.7+x}[{\mathrm{Fe}(\mathrm{CN})}_{6}]$$

By comparison, the galvanostatic discharge curves associated with the MZHCF-10 cathode exhibit noticeably smoother sloping profiles spanning 1.8–1.4 V (versus the Zn^2+^/Zn reference) and 1.4–0.9 V (Figure 4d), reflecting a non-abrupt, continuous insertion of zinc-ion carriers guided by solid-solution thermodynamics. Corroborating the cyclic voltammetry analysis, these distinct electrochemical slopes are directly associated with the faradaic redox activities of the manganese-based (Mn^2+/3+^) along with iron-based (Fe^2+/3+^) couples, respectively. Under a current load of 0.5 A g^−1^, the pristine ZnHCF electrode yields a specific output of 48.7 mAh g^−1^, whereas the manganese-substituted MZHCF-10 achieves a significantly elevated discharge capacity of 150.3 mAh g^−1^. Upon escalating the rate to 1 A g^−1^, both systems preserve a substantial portion of their capacity, exhibiting retention values of 66.3% and 67.8% for ZnHCF and MZHCF-10, respectively, compared to their corresponding metrics at the 0.5 A g^−1^ baseline. The energy density of MZHCF-10 was evaluated to further assess its practical storage capability. Based on the average discharge voltage of 1.5 V, MZHCF-10 delivers a high specific energy density of 225.5 Wh kg^−1^ at 0.5 A g^−1^. This performance remains robust even at 1 A g^−1^ (150.0 Wh kg^−1^), highlighting the efficiency of the Mn-Zn synergy in optimizing both capacity and working voltage.

Long-term electrochemical durability of unmodified ZnHCF, bimetallic MZHCF derivatives, and pure MnHCF was assessed under an applied current load of 1 A g^−1^ (Figure 4e). The pristine ZnHCF electrode exhibited an initial discharge capability of 91.93 mAh g^−1^, which subsequently suffered a rapid and unrelenting decay over successive charging–discharging loops. At the 500th cycle milestone, its specific capacity fell to 51.95 mAh g^−1^ (representing a mere 56.5% of its starting value). Extending the durability test to 2000 cycles triggered a severe capacity plunge down to 33.08 mAh g^−1^, corresponding to an unsatisfactory retention efficiency of only 36%. This severe degradation is closely coupled with a highly destructive cubic-to-rhombohedral phase transformation during faradaic processes, which aggressively accelerates the dissolution of the host matrix into the surrounding aqueous medium. While the macroscopic dissolution of ZnHCF was partially mitigated by the solubility equilibrium established within the concentrated ZnSO_4_ liquid medium, minor leakage of free [Fe(CN)_6_]^3−^ complex ions persists. These detached anions can migrate toward the metallic zinc anode to instigate parasitic side reactions, or alternatively, coordinate with dissociated Zn2+ cations in the liquid phase to form inactive precipitates. Consequently, the host material undergoes catastrophic capacity fade, rendering it unsuitable for realistic high-performance energy storage platforms.

On the other hand, the MnHCF counterpart displayed an initial discharge capability of 91.24 mAh g^−1^, but its electrochemical trajectory during cycling followed a highly non-monotonic wave-like path involving an initial decay, a subsequent activation-induced rise, and a final gradual deterioration. Specifically, the capacity dropped to 66.8 mAh g^−1^ by the 62nd cycle, progressively climbed back to a peak value of 82.35 mAh g^−1^ after 426 cycles of structural electro-activation, and eventually settled at 69.23 mAh g^−1^ at the 2000-cycle termination point, which corresponds to a retention efficiency of 75.9%. Visually, the clear liquid medium turned pale yellow over the course of electrochemical testing, revealing that substantial dissolution of the MnHCF host occurs during the early cycles. This confirms that pristine MnHCF lacks the necessary electrochemical stability within aqueous zinc-ion environments.

Remarkably, the bimetallic MZHCF configurations demonstrated vastly superior cycling durability relative to both single-metal endmembers. Following 2000 continuous loops, the specific discharge capacities of MZHCF-5, -10, -15, and -20 were measured at 42.36, 106.73, 60.09, and 44.58 mAh g^−1^, respectively. Among this series, the optimal MZHCF-10 configuration displayed unrivaled capacity retention, showing virtually zero deterioration compared to its primary discharge state. When benchmarked against state-of-the-art Prussian blue analogue (PBA) cathodes documented in literature for zinc-ion systems, the MZHCF-10 framework exhibits outstanding lifespan characteristics alongside highly competitive specific capacities (Table S2). To elucidate the underlying structural evolution and charge-transfer mechanisms driving this exceptional stability, ex situ XRD and XPS investigations were carried out on electrodes harvested at selected states of charge/discharge, which are thoroughly discussed in the subsequent subsection.

### 3.3. Zinc-Ion Intercalation Mechanism

**Figure 5:**
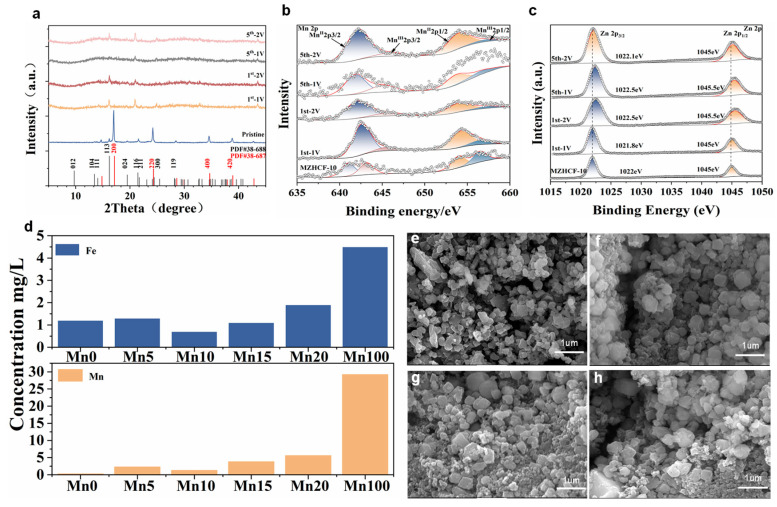
Ex situ XRD of the MZHCF-10 (**a**) electrodes at different states. Mn 2p (**b**) and Zn 2p (**c**) Ex situ XPS spectra of the MZHCF-10 ICP of electrolyte after 2000 cycles (**d**), SEM images of the MZHCF-10 after 0 (**e**), 50 (**f**), 100 (**g**) and 200 (**h**) cycles.

Ex situ X-ray diffraction (XRD) profiles of the MZHCF-10 cathode, collected at designated electrochemical states under a low galvanostatic rate of 50 mA g^−1^, are displayed in Figure 5a. In its fresh, uncycled state, the MZHCF-10 matrix manifests a hybrid crystallographic nature comprising coexisting rhombohedral and cubic symmetries. When polarized to a fully discharged potential of 1.0 V, the characteristic cubic reflections indexed to the (200), (400), and (420) lattice planes vanish, whereas the corresponding (220) signal undergoes severe attenuation. Concurrently, the shifting trends and amplitude variations of the rhombohedral diffraction signals precisely replicate those observed within the unformulated powder diffractogram. Following subsequent extraction back to a charged limit of 2.0 V, the structural lattice fails to restore its pristine configuration. Notably, the cubic (200) reflection remains completely absent, and the rhombohedral peak positions preserve their localized positions from the preceding intercalated state. Upon extending the test to five continuous cycles, the diffractograms of both the oxidized and reduced electrodes develop high structural similarity, locking exclusively into a stabilized rhombohedral framework. Nevertheless, the rhombohedral (113) reflection displays highly reversible behavior, exhibiting systematic attenuation during insertion and subsequent regeneration to its pristine intensity upon extraction. These crystallographic tracking results demonstrate that the catastrophic cubic-to-rhombohedral phase mutation is effectively bypassed through the strategic incorporation of manganese cations. This structural lock-in directly rationalizes the smooth, single-phase solid-solution kinetics evidenced by the electrochemical profiles. During repetitive faradaic cycling, severe periodic lattice reconstruction and phase fluctuations typically accelerate active material dissolution into the liquid phase, triggering rapid performance decay. Consequently, mitigating this phase boundary evolution is paramount to reinforcing the overall structural integrity of the coordination framework, culminating in a vastly extended service lifetime.

Ex situ XPS characterization was performed on MZHCF-10 (Figure 5b,c). In the first cycle, upon discharge to 1.0 V, the binding energy of Mn^3+^ increases while that of Mn^2+^ decreases; upon charge to 2.0 V, the binding energy of Mn^3+^ decreases while that of Mn^2+^ increases. This indicates that Mn ions participate in the electrochemical process of ZnHCF, being oxidized to Mn^3+^ at the fully charged state and reduced to Mn^2+^ at the discharged state. Notably, the opposite trend is observed in the fifth cycle, which may be attributed to the insertion of Mn ions from the electrolyte. The binding energy of Zn 2p gradually increases with cycling, indicating that Mn substitution can enhance the structural stability of ZnHCF, suppress the phase transition process, and improve the discharge capacity and cycling stability.

We also measured the concentrations of Fe and Mn ions in the electrolyte after 2000 cycles for ZnHCF, MZHCFs, and MnHCF (Figure 5d). The results show that, among all samples, the electrolyte from MZHCF-10 exhibits the lowest concentrations of both Fe and Mn ions, indicating that a Mn content of 10% minimizes the dissolution of the cathode material to the greatest extent, thereby providing the most significant enhancement in electrochemical performance. Furthermore, the SEM images of MZHCF-10 after 0, 50, 100, and 200 cycles (Figure 5e–h) exhibit virtually no change in morphology, further confirming the excellent stability and cycling performance of MZHCF-10.

The transition from a two-phase reaction to a solid-solution mechanism is the cornerstone of the improved performance in MZHCF-10. In pristine ZnHCF, the discrete cubic-rhombohedral transition creates significant mechanical stress and favors material dissolution. In contrast, the Mn-induced solid-solution pathway facilitates continuous Zn^2+^ diffusion without the formation of resistive phase boundaries. Furthermore, the stabilization of the water-rich cubic phase provides a more robust environment for reversible redox activity, as supported by our DOS and ex situ XRD analyses. This multifaceted synergy ensures that MZHCF-10 can withstand long-term cycling while maintaining rapid ion-transport kinetics.

## 4. Discussion

To conclude, manganese-doped zinc hexacyanoferrate (MZHCF) frameworks were successfully fabricated through a facile room-temperature wet-chemical precipitation protocol to enhance the electrochemical durability of aqueous zinc-ion batteries. Elemental and crystallographic evaluations verified that the incorporated manganese fraction remained relatively dilute, thereby inducing no prominent spectroscopic modifications. Nevertheless, the concurrent occupancy of both transition-metal species within the nitrogen-coordinated nodes triggered substantial alterations in their charge-storage pathways. This multi-cation coordination architecture established a cooperative synergy that dramatically bolstered cycling stability, culminating in capacity retention capabilities that far outstrip those of single-metal endmembers (ZnHCF and MnHCF). Specifically, the MZHCF-10 configuration showcased the most outstanding durability; after undergoing 2000 continuous galvanostatic loops at an applied current load of 1 A g^−1^, its specific discharge capacity showed virtually zero degradation, presenting a stark contrast to the pristine ZnHCF matrix, which preserved a mere 36% of its initial value. The unprecedented electrochemical stability of MZHCF-10 stems from a multi-level synergistic effect between manganese and zinc. Structurally, the Mn-Zn coexistence tailors the N-coordinated metal site, favoring a solid-solution reaction pathway that suppresses the detrimental cubic-to-rhombohedral phase transition characteristic of pristine ZnHCF. Chemically, the presence of Zn ions stabilizes the framework against Jahn–Teller distortion, while Mn ions minimize the dissolution of the active material into the aqueous electrolyte. This dual-protection mechanism, supported by our ICP results and DOS calculations, ensures the structural integrity of the open-framework host throughout 2000 cycles. The nitrogen-linked metallic nodes within Prussian blue frameworks exert a profound influence on dictating overall electrochemical durability. This systematic investigation establishes that strategically adjusting the nitrogen-linked transition-metal configuration offers a powerful approach to fine-tuning divalent zinc-ion storage capabilities.

## Figures and Tables

**Table 1:** Compositions of the ZnHCF and MZHCFs.

Sample	Formula
ZnHCF	K_0.06_Zn_1.71_[Fe(CN)_6_]
MZHCF-5	K_0.05_Mn_0.05_Zn_1.73_[Fe(CN)_6_]
MZHCF-10	K_0.07_Mn_0.09_Zn_1.69_[Fe(CN)_6_]
MZHCF-15	K_0.16_Mn_0.13_Zn_1.66_[Fe(CN)_6_]
MZHCF-20	K_0.10_Mn_0.14_Zn_1.59_[Fe(CN)_6_]

## Data Availability

The original contributions presented in this study are included in the article/Supplementary Materials. Further inquiries can be directed to the corresponding authors.

## Supplementary Materials

The following supporting information can be downloaded at: https://www.mdpi.com/article/10.3390/nano16100617/s1, Figure S1. Fe 2p XPS spectra of the ZnHCF, and MZHCF-10 samples. Figure S2. SEM images of the MZHCF-5 (a), MZHCF-15 (b), and MZHCF-20 (c) samples. EDS maps (d) for K, Mn, Zn, Fe, C, N, and O of MZHCF-10. Figure S3. CV curve s of ZnHCF (a) different samples at a scan rate of 1 mV s^−1^ (b). Galvanostatic charge/discharge (GCD) curves of ZnHC at different current densities (c). Figure S4. Cycling curves of ZnHCF (a) and MZHCF-10 (b) in different electrolytes. Figure S5. Electrochemical impedance spectroscopy of ZnHCF and MZHCFs. Table S1. The elemental ratios of K, Zn, Fe, and Mn in ZnHCF and MZHCFs. Table S2. Comparison of the performance of MZHCF-10 with previously reported PBAs. Refs. [44,46,53,54,55,56,57,58,59,60] are cited in the supplementary materials.

## Abbreviations

The following abbreviations are used in this manuscript:

AZIBs	Aqueous zinc-ion batteries
PBAs	Prussian blue analogues
ZnHCF	zinc hexacyanoferrate
MZHCFs	zinc in manganese-substituted zinc hexacyanoferrates
NMP	N-methylpyrrolidone
CV	Cyclic voltammetry
DOS	density of states
DI	deionized water
