# Supplementary material for: Manganese–Zinc Synergy in Prussian Blue Analogues for Long-Cycle Aqueous Zinc-Ion Battery Cathodes
Source: Nanomaterials (Basel). 2026 May 17;16(10):617. doi: 10.3390/nano16100617 (PMC13209455; doi:10.3390/nano16100617)
Supplement: Supplementary file 1 [file nanomaterials-16-00617-s001.zip › nanomaterials-4314999-supplementary.pdf]

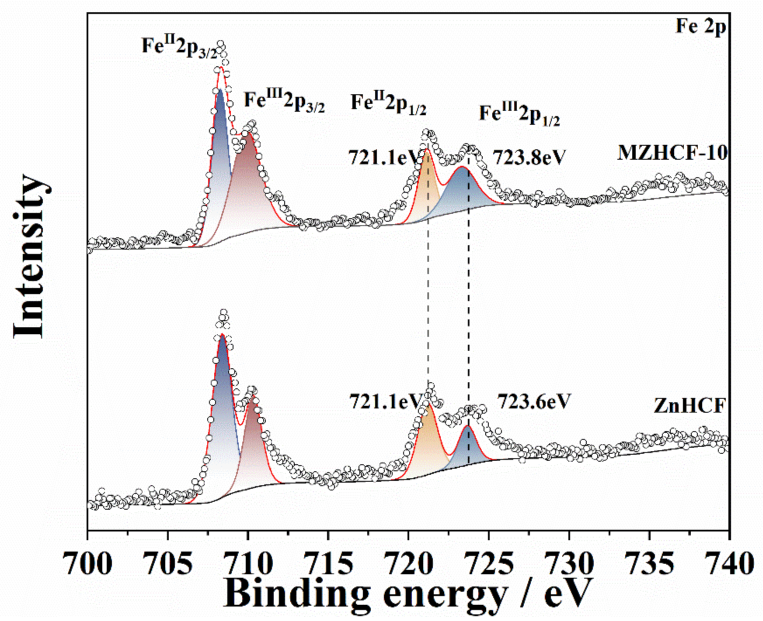

Figure S1. Fe 2p XPS spectra of the ZnHCF, and MZHCF-10 samples.

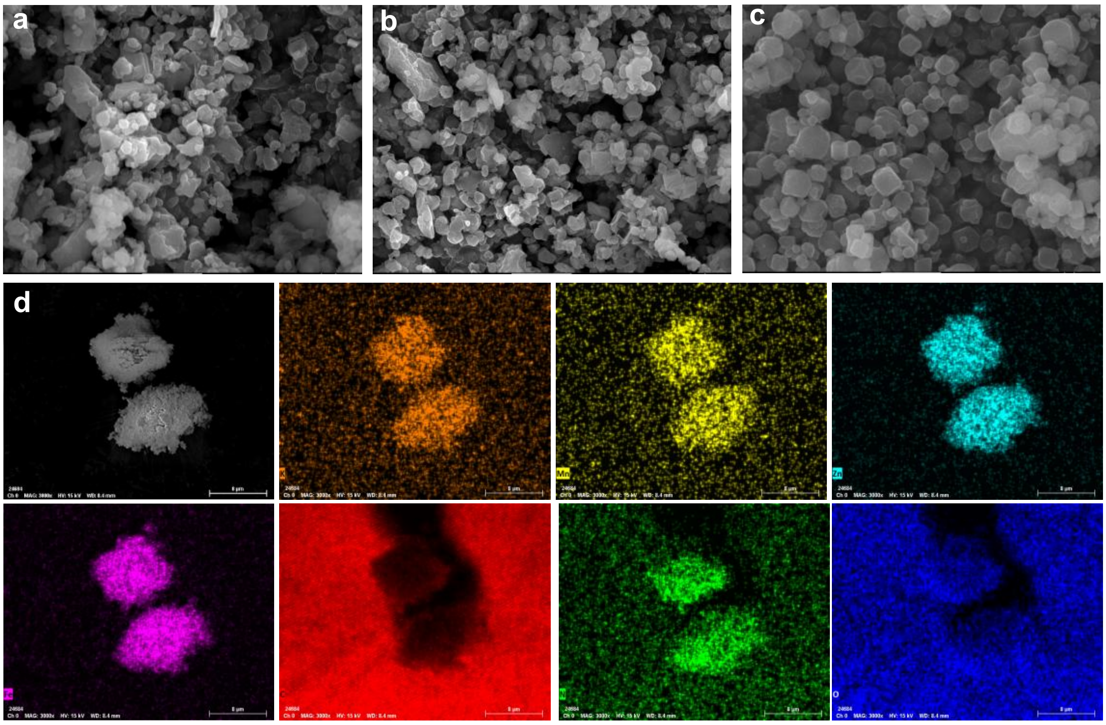

Figure S2. SEM images of the MZHCF-5 (a), MZHCF-15 (b), and MZHCF-20 (c) samples. EDS maps (g) for K,Mn,Zn, Fe, C ,N,and O of MZHCF-10.

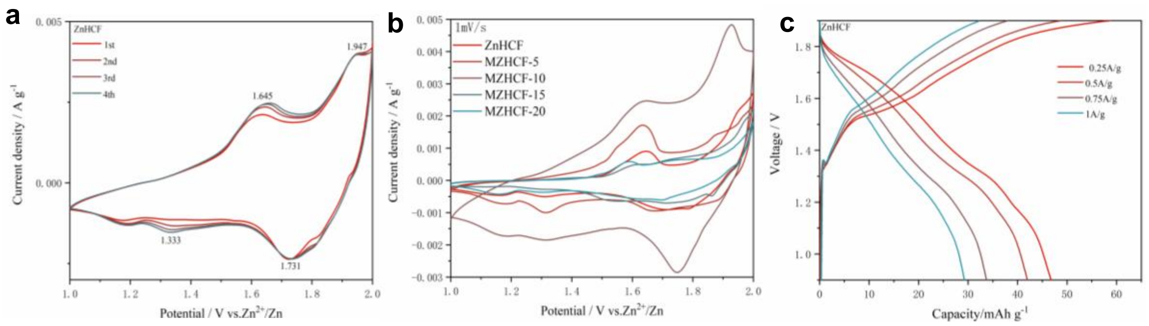

Figure S3.CV curves of ZnHCF(a) different samples at a scan rate of 1mV s^{-1} (b). Galvanostatic charge/discharge (GCD) curves of ZnHC at different current densities(c).

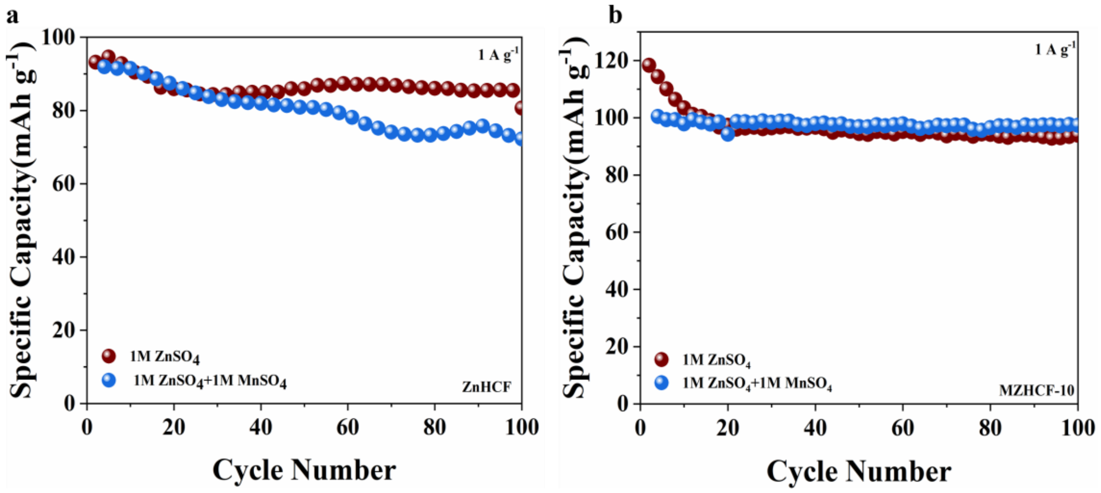

Figure S4. Cycling curves of ZnHCF(a) and MZHCF-10(b) in different electrolytes

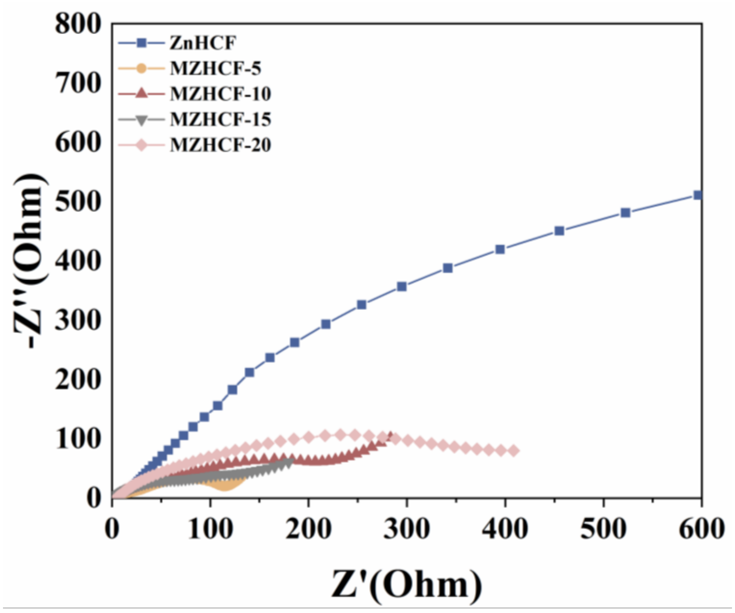

Figure S5 Electrochemical impedance spectroscopy of ZnHCF and MZHCFs

Table S1. The elemental ratios of K, Zn, Fe, and Mn in ZnHCF and MZHCFs

ZnHCF	Mass fraction(%)
Fe	14.9209
K	0.8567
Zn	25.5838
MZHCF-5	Mass fraction(%)
Fe	12.5951
K	0.6041
Mn	0.6636
Zn	21.8502
MZHCF-10	Mass fraction(%)
Fe	10.5781
K	0.6958
Mn	0.9604
Zn	17.9103
MZHCF-15	Mass fraction(%)
Fe	12.2563
K	1.9095
Mn	1.6348
Zn	20.3822
MZHCF-20	Mass fraction(%)
Fe	15.1245
K	1.5648
Mn	2.1503
Zn	24.0727

Table S2. Comparison of the performance of MZHCF-10 with previously reported PBAs

Cathode material	Current density	Specific capacity (mAh g^{-1})	Cycle life (n)	Capacity retention rate (%)
ZnHCF[1]	1C	65.4	100	76
ZnHCF[2]	0.1C	70		
CoMn-PBA HSs[3]	1 A g^{-1}	55.7	1000	76.4
$Mn_{0.8}Zn_{0.2}HCF$[4]	0.85 A g^{-1}		1000	73.2
ZnHCF@MnO_2[5]	500 mA g^{-1}	90	1000	77
ZnHCF/PANI[6]	0.5 A g^{-1}	100	300	75.2

HE-PBA[7]	0.1 A g^{-1}	89	100	82
CuHCF[8]	1C	58	400	49
$Na_2Mn[Fe(CN)_6]\bullet_{2.78}H_2O$[9]	50 mA g^{-1}	53.7	120	60
NiHCF[10]	0.1 A g^{-1}	73.9	1000	75
This work	1 A g^{-1}	100	2000	~100

References

1. Zhang, L.; Chen, L.; Zhou, X.; Liu, Z. Towards High-voltage Aqueous Metal-ion Batteries beyond 1.5 V: The Zinc/Zinc Hexacyanoferrate System. *Advanced Energy Materials* **2015**, *5*, 1400930, doi:10.1002/aenm.201400930.
2. Kim, D.; Lee, C.; Jeong, S. A Concentrated Electrolyte for Zinc Hexacyanoferrate Electrodes in Aqueous Rechargeable Zinc-Ion Batteries. *IOP Conf. Ser.: Mater. Sci. Eng.* **2018**, *284*, 012001, doi:10.1088/1757-899X/284/1/012001.
3. Zeng, Y.; Lu, X.F.; Zhang, S.L.; Luan, D.; Li, S.; Lou, X.W. (David) Construction of Co–Mn Prussian Blue Analog Hollow Spheres for Efficient Aqueous Zn-Ion Batteries. *Angewandte Chemie International Edition* **2021**, *60*, 22189–22194, doi:10.1002/anie.202107697.
4. Pan, Z.-T.; Li, B.; Xu, Y.; Kong, L.-B. Zn-Substituted MnHCF Suppresses the Jahn–Teller Distortion and Enhances Ionic Conductivity. *Journal of Electroanalytical Chemistry* **2026**, *1007*, 119923, doi:10.1016/j.jelechem.2026.119923.
5. Lu, K.; Song, B.; Zhang, Y.; Ma, H.; Zhang, J. Encapsulation of Zinc Hexacyanoferrate Nanocubes with Manganese Oxide Nanosheets for High-Performance Rechargeable Zinc Ion Batteries. *J. Mater. Chem. A* **2017**, *5*, 23628–23633, doi:10.1039/C7TA07834J.
6. A Polyaniline Surface-Modified Prussian Blue Analogue Cathode for Flexible Aqueous Zn-Ion Batteries. *Chemical Communications* **2022**, *58*, 8226–8229, doi:10.1039/d2cc02724k.
7. Xing, J.; Zhang, Y.; Jin, Y.; Jin, Q. Active Cation-Integration High-Entropy Prussian Blue Analogues Cathodes for Efficient Zn Storage. *Nano Res.* **2023**, *16*, 2486–2494, doi:10.1007/s12274-022-5020-0.
8. Kasiri, G.; Trócoli, R.; Bani Hashemi, A.; La Mantia, F. An Electrochemical Investigation of the Aging of Copper Hexacyanoferrate during the Operation in Zinc-Ion Batteries. *Electrochimica Acta* **2016**, *222*, 74–83, doi:10.1016/j.electacta.2016.10.155.
9. Li, W.; Xu, C.; Zhang, X.; Xia, M.; Yang, Z.; Yan, H.; Yu, H.; Zhang, L.; Shu, W.; Shu, J. Sodium Manganese Hexacyanoferrate as Zn Ion Host toward Aqueous Energy Storage. *Journal of Electroanalytical Chemistry* **2021**, *881*, 114968, doi:10.1016/j.jelechem.2020.114968.

10. Wang, K.; Xu, Z.; Li, H.; Wang, H.; Ge, M.; Liu, J.; Li, S.; Hu, Z.; Zhu, M.; Zhang, Y.; et al. Realizing the Highly Reversible Zn2+ and Na+ Dual Ions Storage in High-Crystallinity Nickel Hexacyanoferrate Microcubes for Aqueous Zinc-Ion Batteries. *Journal of Materials Science & Technology* **2023**, *164*, 102–110, doi:10.1016/j.jmst.2023.04.023.
